# Unpacking the Emotional Dimension of Doctoral Supervision: Supervisors' Emotions and Emotion Regulation Strategies

**DOI:** 10.3389/fpsyg.2021.651859

**Published:** 2021-06-28

**Authors:** Ye Han, Yueting Xu

**Affiliations:** ^1^School of Humanities and Social Sciences, Harbin Institute of Technology Shenzhen, Shenzhen, China; ^2^Center for Linguistics and Applied Linguistics, Guangdong University of Foreign Studies, Guangzhou, China

**Keywords:** doctoral supervision, supervisor, emotion, emotion regulation, higher education

## Abstract

Successful completion of a PhD is challenging for both the candidate and the supervisor. While doctoral students' emotional burdens received much attention, their supervisors' emotional experiences remain under-explored. Moreover, while teacher education research stressed the importance of teacher emotion regulation, empirical studies on doctoral supervisors' emotion regulation barely exist. The current qualitative study explored 17 computer science supervisors' emotions unfolding in doctoral supervision and their emotion regulation strategies. Semi-structured interviews revealed the supervisors' wide-ranging emotions, with their negative emotions more diverse and common than positive ones. The supervisors also regulated their emotions through multiple strategies within antecedent-focused and response-focused approaches. As one of the initial studies on doctoral supervisors' emotion and emotion regulation in their own right, the current study not only uncovers the complexity of the emotion-laden dimension of supervision, but also highlights the need for all stakeholders to attend to supervisors' psychological well-being in tandem with their students'.

## Introduction

Doctoral supervision is rewarding but challenging experiences for academics (Elliot and Kobayashi, 2019). Even without sufficient professional training (Acker and Haque, 2015), supervisors are expected to balance conflicting roles and to meet individual students' needs (Hemer, 2012; Benmore, 2016) in contemporary higher education marked with growing managerialism, performativity, and accountability (Aitchison et al., 2012). These demands can induce supervisors' anxiety (Halse, 2011), frustration (Robertson, 2017) and exhaustion (González-Ocampo and Castelló, 2019), which suggests the need for supervisors to self-regulate emotions. In the neighboring field of teacher development, teacher emotions have been found to influence teaching and learning (Sutton and Wheatley, 2003). Similar anecdotal evidence also appeared in doctoral supervision literature (Sambrook et al., 2008). However, despite increasing attention to psychological well-being of doctoral students (Cotterall, 2011; Acker and Haque, 2015; Virtanen et al., 2017), supervisors' emotional experiences remain underrepresented. To address this research gap, we reported a qualitative study as an initial attempt that looks into doctoral supervisors' emotions and emotion regulation in their own right.

## Background Literature

### Emotion and Emotion Regulation

Once considered inferior to cognition, emotion has been gaining momentum in education, psychology, and other fields of social science in past decades. Although definitional complexity still lingers, emotion is conceptualized as multifaceted and dynamic, involving physiological, psychological, cognitive, behavioral, and affective responses (Frenzel and Stephens, 2013) to situations appraised as meaningful to individuals or groups (Lazarus, 1991).

Building on this conceptualization, Gross (1998, 2015) has proposed the process model of emotion regulation. In this model, emotion regulation was conceived as one's conscious or unconscious effort to influence what, when, and how emotions occur, are experienced, and expressed. Although emotion regulation often aims to reduce negative emotions and/or enhance positive emotions, counterhedonic emotion regulation also exists, i.e., worsening one's emotional experiences (Gross, 2015, p. 5).

In terms of the target of emotion regulation, one can engage with intrinsic emotion regulation (influencing his/her own emotions, also termed as *intrapersonal emotion regulation*, Niven, 2017) or extrinsic (influencing others' emotions, also termed as *interpersonal emotion regulation*, Niven, 2017) (Gross, 1998). Intrinsic and extrinsic emotion regulation can be closely related. First, a single regulatory strategy may be both intrinsic and extrinsic simultaneously (Gross, 2015; Guan and Jepsen, 2020). More important, intrinsic emotion regulation can be achieved through seeking extrinsic emotion regulation from others. This strategy was identified as *intrinsic interpersonal emotion regulation* by Zaki and Williams (2013). Altan-Atalay and Saritas-Atalar (2019) further suggested that this strategy (more specifically, having others assure oneself that the situation was under control) particularly benefits individuals who lack confidence in intrinsic emotion regulation.

Regardless whose emotions one intends to regulate, emotion regulation can be realized through two broad approaches: (a) antecedent-focused approach, which alters factors inducing emotions to avoid or modulate emotions; and (b) response-focused approach, which changes one's emotional responses and expressions after an emotion fully blossoms. Each approach consists of several strategies (see Table 1).

**Table 1:** Approaches and strategies of intrinsic emotion regulation adapted from Gross (1998, 2015) and Zaki and Williams (2013).

**Emotion regulation approaches**	**Emotion regulation strategies**	**Definition**
Antecedent-focused	Situation avoidance	Avoiding situations likely to induce particular emotions
	Situation modification	Changing or manipulating situations to invoke, strengthen, or reduce particular emotions
	Cognitive change	Reappraising or reinterpreting situations to alter emotions
	Attention deployment	Distracting one's own attention from emotions and emotion-invoking situations
	Use of extrinsic emotion regulation	Consulting others to obtain an assurance that the emotion-eliciting situation is under control and solutions can be devised
Response-focused	Relaxation techniques	Using relaxation techniques to change one's emotional responses to a situation
	Suppression	Controlling oneself from showing and/or experiencing an emotion

The importance of emotion and emotion regulation has long been recognized in the neighboring field of teacher development. Positive emotions were found to enhance teacher-student relationship, promote flexible and creative teaching, strengthen students' motivation (Frenzel et al., 2009) and their learning (Hargreaves, 1998), whereas negative emotions reverse these effects (Sutton and Wheatley, 2003), which indicates the necessity of teacher emotion regulation.

Teacher emotion regulation has mainly been investigated under two frameworks: emotion labor (Hochschild, 1983) and emotion regulation (Taxer and Gross, 2018). While emotion regulation is at core of emotion labor (Alam et al., 2019), two frameworks have different emphases. Emotion labor highlights one's display of emotions according to sociocultural, sociopolitical, institutional and organizational rules and expectations (Hochschild, 1983), whereas emotion regulation does not emphasize whether the emotions are to be shown in relation to rules.

Although doctoral supervision is different from classroom teaching, it does include a pedagogical dimension (Cotterall, 2011). The crucial role of emotion and emotion regulation is thus likely to hold true in doctoral supervision. In fact, Sambrook et al. (2008) has reported evidence that a doctoral supervisor was reluctant to regulate students' negative emotions before she successfully regulated her own. Nonetheless, empirical research on supervisors' intrinsic emotion regulation is very rare. It is thus necessary to explore this matter to pave the way for future research into the relationship between supervisors' emotions, emotion regulation, sociocultural and institutional requirements.

### Challenges to Doctoral Supervision in Relation to Supervisors' Emotional Experiences

Doctoral supervision is highly complex and demanding, which may take a heavy toll on supervisors' emotions. Supervisors need to balance myriad contradictory responsibilities and roles, such as developing students' autonomy vs. helping them reach academic milestones efficiently (Overall et al., 2011; Aitchison et al., 2012). Another challenge is to meet the needs of different students, and of the same student at different stages of candidature (Deuchar, 2008; Benmore, 2016). Moreover, global moves toward managerialism and accountability trickle down to day-to-day pressure on both supervisors and students to be productive (Aitchison et al., 2012). In addition, supervisor development programs remain limited in many parts of the world (e.g., Acker and Haque, 2015). Many academics apply their own experience being a doctoral student (Hemer, 2012; González-Ocampo and Castelló, 2019) or experience advising undergraduate and master's students to supervising doctoral students (Halse, 2011).

Given the complexity of doctoral supervision, supervisors unsurprisingly experience rich and mixed emotions. They reported positive emotions, such as intellectual and pedagogical pleasures (Halse and Malfroy, 2010) and love (Deuchar, 2008). However, negative emotions were more common, including frustration, anger (Aitchison et al., 2012), anxiety (Halse, 2011), exhaustion (González-Ocampo and Castelló, 2019), guilt (Halse, 2011), frustration (Robertson, 2017), and disappointment (Sambrook et al., 2008). To cope with these emotions, supervisors were found to use response modulation (e.g., lowering one's voice and slowing down to calm oneself, Halse, 2011), and cognitive change, (e.g., reframing the student's failure as the supervisor's fault, Sambrook et al., 2008), but some also struggled to walk out of negative emotions (Sambrook et al., 2008). Nonetheless, empirical studies on doctoral supervisors' intrinsic emotion regulation remain scarce.

In sum, although researchers have emphasized the emotional aspect of teaching and highlighted emotional challenges of supervising doctoral students, little is known about doctoral supervisors' emotions and their emotion regulation strategies. The dearth of research becomes even more glaring in the Chinese context, of which doctoral education started late but expands rapidly (Zheng et al., 2018). A qualitative study was thus conducted to answer two research questions:

- What emotions do the supervisors experience when supervising doctoral students?
- Do they regulate their own emotions? If so, what strategies do they use?

## Methods

### Participants

Unlike their international counterparts, academics in China only become eligible for supervising doctoral students when (a) their institutions are approved by the Ministry of Education for offering doctoral programs, and (b) they have met multiple institutional requirements, especially in terms of high-quality publications and research funding. Therefore, doctoral supervisors in China are an elite group of full professors (and very rarely, associated professors), mostly working in prestigious institutions and being limited in number. Considering these contextual constraints, we recruited participants through convenience sampling in combination with snowball sampling. We approached an acquaintance, a computer science doctoral supervisor in a key institution, introduced our research aim and clarified participants' right. After he agreed to participate, we requested him to introduce his colleagues to us. We also sent out invitations to computer science doctoral supervisors in other institutions through email and instant messaging APPs. Partly adopting Åkerlind and McAlpine's (2017) sampling rationale, we made efforts to ensure participants' variations in terms of prestige of institutions, experience as a supervisor, region, and gender. Out of 32 supervisors being approached, 17 consented to participate, whose background is shown in Table 2. They are anonymized as Participant 1, 2, ….17, and abbreviated as P1, P2…P17 in this paper.

**Table 2:** Demographic characteristics of the participants.

**Characteristics**		***N***
Total (*N*)		17
Gender	Male	13
	Female	4
Region	Northern	5
	Central	2
	Eastern	3
	Southern	7
Prestige of HE institutions	Key institutions	14
	Regional institutions	3
Career stage	Novice (doctoral graduate = 0, and doctoral supervision experience ≥1 year)	4
	Mid-career (doctoral graduate ≥1, and doctoral supervision experience ≤10 years)	11
	Veterans (doctoral graduate ≥10, and doctoral supervision experience ≤10 years)	2

### Data Collection

The data were collected through individual semi-structured interviews. Since the researchers and the participants were working from home due to the Covid-19 pandemic in the spring of 2020, all interviews took place through phone calls or Wechat audio calls. Each interview was between 30 and 90 min, during which the participants were asked to describe their general perceptions of doctoral supervision, their supervisory style, their emotions unfolding in doctoral supervision, and whether and how they regulated their own emotions. Probing questions were raised when relevant, surprising, and ambiguous responses were provided to gain an in-depth understanding of the doctoral supervisors' emotional experiences and their intrinsic emotion regulation strategies. The interview guide is presented in Appendix. These interviews were delivered in Chinese (all participants' first language), audio-recorded, and transcribed verbatim.

### Data Analysis

To analyze data, we firstly read transcripts repeatedly to identify and label strings of texts relevant to research questions mainly based on participants' original words. For instance, P1 constantly described himself feeling “nervous,” thus relevant excerpts were labeled as “nervousness/hecticness.” The initial codes were constantly revised as informed by literature (e.g., Pekrun and Linnenbrink-Garcia, 2012; Rowe et al., 2014, p. 286, based on Lazarus, 2006) and ground on the current data. For instance, in this process, initial code “nervousness/hecticness” was further subsumed to “anxiety,” operationalized as the feelings aroused by an anticipation of an uncertain, pending threat or failure (Pekrun and Linnenbrink-Garcia, 2012; Rowe et al., 2014). Meanwhile, we also created case summaries and memos to capture the characteristics of each supervisor's emotional experiences. The finalized categories of emotions involved three valence-based classifications: positive, negative, and mixed emotions, and the first two of which were broken down into a number of discrete emotions. Regarding the emotion regulation strategies, two broader approaches, i.e., antecedent-focused and response-focused, emerged from the data. The former included situation selection, situation modulation, cognitive change, attention deployment. Since three participants also purposefully communicated with others to alter their own emotions, *seeking extrinsic emotion regulation* was added, which was not included in Gross's (1998, 2015) framework. The response-focused approach included suppression and relaxation. In other words, the revised emotion regulation was consistent with Table 1 introduced earlier in the literature review.

To enhance credibility and trustworthiness of data analysis, the second author coded 50% of the data and yielding an initial inter-coder agreement of 85%. The inconsistent codes were discussed after consulting previous literature and finally resolved. Interview summaries and findings were also confirmed by four participants, who were willing to attend a member-checking process.

## Findings

### Doctoral Supervisors' Emotion Experience

The interviews indicated that doctoral supervisors experienced a variety of emotions in doctoral supervision, both positive and negative. However, positive emotions were less mentioned and less diverse than negative emotions. In addition, two participants (P7 & P8) also described rather mixed emotions in doctoral supervision: “I felt everything, joy, anger, sadness, and happiness. It's quite mixed” (P7). Table 3 summarizes the participants' emotions.

**Table 3:** The participants' emotions unfolding in doctoral supervision.

**Valence**	**Discrete emotions**	**Participants (*n*)**	**Data extracts (*n*)**
Positive	Happiness	11	18
	Love^1^	4	6
Negative	Anger	12	17
	Anxiety	7	13
	Disappointment	5	9
	Frustration	5	6
	Sadness	3	6
	Exhaustion	3	4
	Guilt	2	2
	Pain	2	2
Mixed	Mixed feelings	2	2

1 *Love means non-romantic, parental-like affection, fondness, and care of students*.

As shown in Table 3, positive emotions included *happiness* and *love*, whereas negative emotions involved 10 discrete emotions. Compared to love, happiness was much more common and consisted of pedagogical pleasure (Elliot and Kobayashi, 2019) and intellectual pleasure (Halse and Malfroy, 2010). Pedagogical pleasure emerged when nine participants witnessed students' growth and achievements, as well as their own growing competence as supervisors (P2 & P14), whereas intellectual pleasure arose when supervisors ignited their motivation and enjoyment in extending their knowledge boundary:

“I feel quite fulfilled in doctoral supervision because this is a learning opportunity for me too. I have a family and am short of energy and time. I'm motivated by my students to keep up with the new knowledge” (P14).

P16 felt joyful as he perceived himself as an increasingly competent supervisor good at cultivating sound supervisory relationship.

“My students often communicate with me proactively. Those moments convinced me that I'm a successful supervisor because students are willing to approach me and talk to me about academic and non-academic issues” (P16).

In addition to happiness, love also emerged from the data. For instance, P8 articulated her fondness of self-directed and competent students:

“We supervisors are really fond of an excellent ‘academic seedling’, who loves doing research, exceeds your expectations, and has his/her own pursuits” (P8).

Love also arose as supervisors and supervisees formed a long-lasting bond along the doctoral process, during which they overcame difficulties together.

“Doctoral research is arduous. In this process, no matter how effective or ineffective the supervisor-supervisee communication is, there is strong emotional attachments to each other” (P13).

Corresponding to our review of literature, the participants experienced a wide spectrum of negative emotions. Within negative emotions, anger was the most prevalent, reported by 12 participants, followed by anxiety (seven participants), frustration (five participants) and disappointment (five participants). Other four negative emotions, i.e., sadness, exhaustion, guilt, and pain, were occasionally mentioned by three or two participants.

Anger was usually ignited when students violated either written or unwritten rules and conventions that supervisors (a) regarded as the cornerstone of a trusting supervisory relationship, and (b) considered as tacit knowledge of both parties. P1 and P12 were furious when their students secretly submitted poorly-written manuscripts without their approval. P4 was irritated because a student missed deadlines repeatedly without informing neither P4 nor other fellow students of his difficulties in doing research. P6 was angry because he received a cursory draft very close to the deadline of an international conference, which left him no choice but burning the night oil to revise the draft for three consecutive days.

The participants' anxiety, however, stemmed unanimously from students' difficulties in conducting research, for instance:

“I had a student who used to major in mechanics, not computer science. His grade was below average. He had some ideas but couldn't realize them, which made me quite anxious” (P4).

Although disappointment was also related to students' research difficulties, it was induced by the dissonance between supervisors' expectations and students' actual achievements or performances. For example, despite his repeated reminders, P6 disappointingly found some students “commit[ting] the same mistakes. I don't understand why some students can be so NOT self-disciplined!” (capitalization added)

While previous research attributed supervisors' frustration to their inadequate competence in supporting students (Aitchison et al., 2012), contextual constraints (Robertson, 2017), and students' misunderstanding (González-Ocampo and Castelló, 2019), four out of five participants who reported frustration linked this emotion to the students' failure to make progress despite supervisors' effort: “Sometimes I got frustrated. I had done so much but (their work) was still not solid” (P11). Frustration was also caused by supervisors' failure to understand students' perspective. After witnessing a student bursting into tears because her work was criticized publicly in a lab meeting, P2 “was rather frustrated” because he didn't understand “why this was worth crying for.” He later added that he lacked confidence in handling students' emotions, which also escalated his frustration.

Sadness emerged when supervisors believed that they were unable to make a difference after being disappointed repeatedly:

“I sometimes feel very upset and sad. Some PhD students' knowledge and skills foundation were so poor that I felt I couldn't do anything about it” (P7).

The study revealed that exhaustion was reported in two scenarios: (a) when supervisors felt overwhelmed by the workload, or (b) when students consistently frustrated supervisors with poor work, which finally added up to exhaustion:

“Some students are not committed to research. After several supervision meetings, they still didn't grasp my idea. I felt exhausted and wanted to cry. Tired. Exhausted” (P2).

Guilt and pain occurred occasionally, with only two participants reporting relevant experiences. Corroborating Halse's (2011) findings, guilt emerged as the supervisors attributed students' unsatisfactory research performance to supervision:

“I often feel guilty because I would question whether I offered sufficient supervision when my student encountered difficulties” (P17).”

P2 and P14 reported feeling painful in supervising doctoral students. P2 regarded his entire experience supervising students as “painful,” whereas P14 specifically linked this emotion to developing student's writing abilities: “Revising students' manuscripts is really painful.” His experience resonated with Aitchison et al.'s (2012) finding that supervisors and supervisees both suffered from the process of learning to write.

### Doctoral Supervisors' Intrinsic Emotion Regulation

The interviews indicated that the participants regulated their emotions induced by doctoral supervision through antecedent-focused approach and response-focused approach. While they mostly aimed to reduce negative emotions, counterhedonic regulation occasionally occurred as well. Table 4 summarizes the participants' intrinsic emotion regulation approaches and strategies.

**Table 4:** Summary of the participants' intrinsic emotion regulation approaches and strategies.

**Approaches**	**Strategies**	**#Cases (*n*)**	**#Data extracts (*n*)**	**Regulating direction**
Antecedent-focused emotion regulation	Situation selection	4	5	Affective-improving
	Situation modulation	10	13	
	Attention deployment	6	10	
	Cognitive change	14	34	Both affective-improving and affective-worsening (counterhedonic)
	Seeking extrinsic emotion regulation	3	4	Affective-improving
Response-focused emotional regulation	Suppression	8	13	Affective-improving
	Relaxation techniques	4	6	

Situation selection referred to supervisors' intentional avoidance to situations where they might experience negative emotions. This strategy was manifested in four supervisors' rigorous recruitment process, aiming to screen out students with whom they might not get along.

“I'm picky when recruiting students. There are multiple rounds of interviews, tests, and trials. I also need to know the candidate's personality. That's why I barely argued with my students. Only after I'm certain that the candidate and I can work together, I'll admit him/her” (P12).

Situation modulation was implemented through changing supervisory practice to regulate supervisors' emotions. Some supervisors became stricter after getting angry and disappointed on a few occasions where students violated the rules or constantly broke their promises. For instance, P1 had his students write down a memo and sign on it after each supervision meeting to confirm that supervisory feedback has been received. P4 was so frustrated by students' repeatedly late submission that he advanced the deadlines. P12 informed his research team of the unpleasant experience of a student who had to defer and resubmit his dissertation, as the student hastily submitted an unpolished draft without the supervisor's approval. P12 also suggested other students learn a lesson from this incident and never do the same.

Contrastingly, P7, P9, and P10 loosened their control and placed more confidence in student autonomy to regulate their own emotions.

“In the first a few years (of being a supervisor), I was very anxious. I met with students every week but they had very little input to share. Later I gradually ‘loosened my grip’. I just focus on the general picture and have them explore the details. Now they approach me proactively, not the other way around. Now I barely am anxious about supervising students” (P10).

Situation modulation was also reflected in lowering supervisors' expectations of students to reduce negative emotions. P2 indicated that he would attend more to those committed to doctoral research and “completely let him/her free,” if the students lacked motivation. P5 stated that he once had high expectations of students, but only found himself becoming more irritable. He thus decided not to “count on” students' output and did more research on his own:

“When I was young, I always aimed at publishing more and more papers. But even though I invested enormous time in (supervising) students, students were still not productive. Shouldn't I be angry? Considering this may ‘break my bowl’ (idiom: jeopardizing his career), I realized that I couldn't count too much on students' research. Then I watched the time I spent (on supervision) and did research myself” (P5).

Attention deployment referred to supervisors' deliberately or unintendedly directing their attention away from the antecedents of their emotions.

“My strategy is not to think about something bad. I think more about research itself, so I'm less likely to worry about students and their personality” (P6).

P12 and P13, on the other hand, redirected their attention because they had too much to do, for instance:

“I have little time to think and worry. Maybe something new comes up very soon. I don't have time to bog in emotions. Yes, I move on” (P13).

Yet the most common emotion regulation strategy that the participants employed was cognitive change. Fourteen out of 17 participants reappraised (a) the meaning of emotions and the antecedents of emotions, (b) the relevance between emotion-invoking situations and their own role as a supervisor and/or as a person with long-term life goals, and/or (c) students' and their own control over student performance (see Table 5).

**Table 5:** Different patterns of cognitive change employed by the participants.

Meaning reappraisal	Reappraising the meaning of emotions	(a) Rationalizing one's own negative emotions emerging in doctoral supervision (P4, P14, & P17) *“The situation that students missed the deadline […] feels like I've arrived at the meeting at the agreed time only found out the other had not prepared at all. It's natural to get pissed off.”* (P4). (b) Devaluing negative emotions, especially anger (P3, P4, P12, & P17) “*I cannot vent my anger towards students because venting leads to a vicious cycle. If I vent, they dare not express their genuine feelings and thoughts in the future”* (P3).
	Reappraising the antecedents of emotions	(a) Magnifying the value of societal and institutional requirements of doctoral supervisors (P15) “*You'll have much fewer complaints if you deeply understand the underlying rationale of every single change in the rules, policies, and requirements related to doctoral supervision*” (P15). (b) Reframing students' inadequate performance as a temporary stage of long-term development (P3 & P8) “*When students don't meet my expectations, I need to make adjustment quickly. I need to accept that students need time to mature*” (P3). (c) Devaluing failures in the process of doing research (P6, P8, P12, & P15) “*It's natural to have up and downs in scientific exploration*” (P12).
Self-relevance reappraisal	Reappraising the relevance between students' poor performance and oneself as a supervisor	^*^Upgrading supervisors' responsibilities for students' temporary failure in doing research (P12, P16, & P17) “*I asked myself if my requirements were too high*” (P16).
	Reappraising the relevance between research supervision and the supervisor's life goals	Downgrading the relevance of students' research to the supervisor' long-term development (P8 & P9) “*Doing research and supervising students is only part of my life*” (P8).
Subjective control reappraisal	Reappraising students' self- control over their performance	Downgrading students' control over their own performance and the “damage” that they had made (P3, P4, P12, & P16). “*Students have their own emotion burdens. They have a lot of work to do. Some have families to support*” (P16). “*He worked hard but just progressed slowly*” (P12). “*What's done is done. I have to accept the reality that he missed the deadline” (P4)*.
	Reappraising supervisors' control over students' performance	(a) Upgrading one's confidence in students' autonomy and abilities (P9, P10, & P14) “*We should trust students. I used to be afraid that they might not have tackled some challenges but they actually made it. Then I believe I should be more confidence in students*” (P14). (b) Upgrading one's subjective control over students' achievement by lowering the expectations for students (P5, P9, & P16) *“Some students just wanted a doctorate as quickly and easily as possible. I lowered my expectations of their academic achievements, so I wouldn't get upset by their mediocre performance*” (P5). (c) Downgrading supervisors' subjective control over students' performance and development by recognizing students' individual differences (P7, P11, P12, & P15) “*I used to believe every student in this prestigious university is excellent and hard-working, but this belief shattered. Not everyone is alike. You have to accept that students are different*” (P7). (d) Downgrading supervisors' subjective control over students' behaviors by recognizing the limited influences of supervisors on supervisees (P11) “*People have their own agenda. Sometimes you can do very little as a supervisor. Your influences aren't always long-lasting*” (P11).

* *The only intrinsic emotion regulation strategy that served the affect-worsening purpose in the current data*.

Table 5 reveals that the participants engage with cognitive change through upgrading or downgrading the meaning of emotions and antecedents of emotions, reappraising the relevance between students' performance, supervision, and supervisors' own life goals, as well as evaluating students' and their own control over students' performance. Interestingly, while cognitive change was predominantly employed to alleviate negative emotions, two exceptional data extracts emerged, in which P16 and P17 self-reprimanded for not offering as much supervision as they hoped to.

Another emotion regulation strategy that emerged in the data was to seek extrinsic emotion regulation, which was not in Gross's (1998, 2015) emotion regulation framework but identified by Zaki and Williams (2013). P11, P13, and P17 intentionally communicated with others to regulate their own emotions. P11 and P13 turned to family members for support, while P17 mentioned that she would talk to colleagues about her emotional encounters in supervising doctoral students.

“My anxiety is alleviated by my family. […] I told my husband about a student's struggle with fulfilling the graduation requirements in time. He said, ‘it's not your responsibility to guarantee that she can graduate. It's her own responsibility.’ I said, ‘all right then”’ (P13).

“I believe it doesn't do any good if I show anger to students, so it's important not to show it. I also talked to colleagues to discuss students' problems as a way to help me feel better” (P17).

P17's account revealed that she employed cognitive change (downgrading the value of anger), seeking extrinsic emotion regulation (communicating with colleagues), as well as suppression (not showing anger to her student). The first two strategies were antecedent-focused, whereas the latter was response-focused. This finding lends support to Taxer and Gross's (2018) argument that multiple strategies and multiple approaches can be employed in tandem.

In fact, P17 was but one of the eight participants who reported suppressing their emotions. They made effort to refrain themselves from losing their temper:

“When I encounter something really irritating, I usually won't get furious immediately. In the past, when I just started supervising students, I used to be less cautious and more short-tempered, but now I tell myself to lump it first no matter what” (P6)

In addition to suppression, relaxation was another response-focused strategy emerging in the data. The participants undertook various relaxation activities to improve their affective experiences:

“When I get frustrated at doing research, my family will have a trip. [We] go sightseeing” (P8)

“I'm extroverted. Bad mood never stays overnight for me. I'd be fully refreshed the next day after a sound sleep. […] I also go fishing once a week as a way to self-regulate my mind” (P14).

## Discussion

The current qualitative study drew upon the perspectives of 17 Chinese doctoral supervisors in computer science through individual interviews to investigate (a) supervisors' emotions unfolding in doctoral supervision, and (b) whether and how they regulated their own emotions. The participants experienced wide-ranging emotions, including positive, negative, and mixed emotions, adding support to the emotion-laden nature of doctoral supervision (e.g., Sambrook et al., 2008; Halse and Malfroy, 2010; Halse, 2011). The participants' positive emotions were mostly happiness, which incorporated pedagogical pleasure (Elliot and Kobayashi, 2019), and less commonly intellectual pleasure (Halse and Malfroy, 2010). The relative prevalence of pedagogical pleasure was expected, as supervision overlaps with teaching in fostering students' growth (Sambrook et al., 2008; Cotterall, 2011). However, intellectual pleasure, albeit only reported by two participants, reveals mutual intellectual growth of both parties. While such intellectual pleasure was reported by supervisors in humanities (Halse and Malfroy, 2010; González-Ocampo and Castelló, 2019), our findings show that computer science supervisors shared the same feelings, which suggests such an emotion can emerge across disciplines.

The emotion of love was the other positive emotion reported by the participants, although this emotion was scarcely documented in the doctoral supervision literature (Deuchar, 2008) but found in feedback research in higher education contexts (e.g., Rowe et al., 2014). Our findings extended the current knowledge by showing that love could stem from supervisors' profound satisfaction of a competent and autonomous student, as well as from a strong bond between a supervisor and a supervisee forged as they jointly overcome obstacles during the doctoral candidature. It is also worth noting three out of four participants who reported love were female, which may suggest that female supervisors may be more likely to experience this emotion in doctoral supervision. Although a gendered perspective on doctoral supervision is beyond the scope of this study, a possible explanation is that female supervisors are under great pressure to live up to the “perfect” role model, i.e., being intelligent and showing care (Hemer, 2012).

In line with previous literature, the study also reported supervisors' negative emotions of higher frequency and greater diversity than their positive emotions, which generate three insights. First, the study expanded the range of antecedents of supervisors' negative emotions. While previous research revealed supervisors' frustration and exhaustion stemming from students' misunderstandings (González-Ocampo and Castelló, 2019), and sadness and disappointment stemming from students' underperformance (Sambrook et al., 2008), the current inquiry supplemented these findings by showing that students' underperformance, limited competence, weak intrinsic motivation, as well as supervisors' heavy workload could all contribute to frustration, sadness, and exhaustion. Furthermore, while some of these antecedents were anticipated or foreseen by our participants, including students' difficulties in research and academic writing (e.g., Aitchison et al., 2012), as well as dissonance between expectations and students' actual research progress (e.g., Sambrook et al., 2008), they were often irritated by unexpected, surprising, or “shocking” obstacles, such as students' violation of rules, protocols, and promises that supervisors had taken granted for.

Second, such wide-ranging antecedents indicate that doctoral supervisors are susceptible to negative emotions, possibly more so than researchers have reported. While Cotterall (2011) argued that a mismatch of expectation and working style between supervisors and supervisees can cause students' anxiety and stress, the current study extends this point by showing how such a mismatch, as reflected in students' violation of taken-for-granted conventions, backfires supervisors' own emotional experiences. It is even more alarming that this mismatch not merely renders the initial stage of doctoral candidature particularly bumpy (Cotterall, 2011). In fact, P12's experience showed that the mismatch may have been concealed but surfaced at a very late stage of doctoral candidature, which can undermine supervisory relationship and jeopardize timely completion of a doctorate.

Furthermore, the data also uncover the interrelationship between negative emotions that supervisors experienced, suggesting that negative emotions, if not regulated effectively in time, may be aggregated and worsened. Our participants' frustration was induced by students' failure to make progress despite supervisors' persistent effort; and if this situation occurred repeatedly, the participants were prone to experience exhaustion and sadness as they might perceive their effortful support futile. Although our participants who reported exhaustion and sadness did not indicate having psychological problems themselves, exhaustion has been argued to contribute to disengagement (Virtanen et al., 2017). Taken together, the nuanced picture of supervisors' emotional experiences not only confirms the challenging and emotion-laden nature of doctoral supervision (e.g., Aitchison et al., 2012), but also highlights the importance of supervisors' intrinsic emotion regulation to their well-being and supervision.

Regarding the second research question, our participants took both antecedent-focused approach and response-focused approach mostly to improve their emotional experiences and very rarely in the reverse direction. Each approach was implemented through multiple strategies. The former involved situation selection, situation modulation, cognitive change, attention deployment, and seeking extrinsic regulation; and the latter included suppression and relaxation. This finding suggests that the supervisors, like teachers in Taxer and Gross's (2018) study, have a variety of emotion strategies at their disposal.

The deployment of situation selection and situation modulation indicated that doctoral supervisors' adjustments of their practice were not solely driven by the goal to foster students' growth but also to enhance their own emotional experiences. Four participants selected students carefully to avoid negative emotions invoked by supervisor-supervisee mismatch in working styles, and 10 participants modulated their supervision practice to experience less anger, anxiety, disappointment, frustration and sadness. Although doctoral supervision research has advocated a “fit” between supervisors and supervisees (e.g., González-Ocampo and Castelló, 2019), this issue was discussed in relation to supervision *per se*, but barely in the light of supervisors' emotional experience. Our data thus supplement this bulk of literature, showing that supervision practice can be fueled by supervisors' psychological needs as well.

While suppression was the most common emotion regulation strategy found in Taxer and Gross's (2018) study on teachers in the US, our participants adopted cognitive change most frequently. This finding was somewhat unexpected because cognitive change was also under-represented in previous doctoral supervision scholarship. Although the literature consistently advised supervisors to understand students' difficult situations and to show empathy (e.g., Overall et al., 2011), this line of argument was mainly proposed to enhance students' experience, satisfaction, and performance. Building on this argument, we highlight the importance of reappraising the meaning, relevance, and subjective control of student progress, particularly appreciating the nonlinearity of research process and students' individual differences, in benefiting doctoral supervisors' subjective experiences.

Another important insight from the participants' deployment of cognitive change is that individuals could modulate their emotions by reappraising not only antecedents of emotions, but emotions themselves. While P15 preempted his complaints by emphasizing the positive implications of policies and requirements regarding doctoral supervision, four participants (P4, P12, P14, & P17) reappraised the meaning of their negative emotions. They saw these negative emotions as natural and reasonable, thus “forgiving” themselves for suffering from anger or frustration. They also reminded themselves that venting would be useless, so as to improve their emotional experiences. Although cognitive change has been framed as antecedent-focused because it addresses on-going emotions rather than fully blossomed emotions (Gross, 1998, 2015), the current data suggested that the line between antecedent-focused and response-focused approaches may be less clear-cut in reality than it is on the conceptual level.

There is also an overlap between seeking extrinsic emotion regulation and other strategies within antecedent-focused approach. P13's husband's remark that the student herself, rather than the supervisor, should be responsible for the completion of a PhD reduced P13's self-relevance of the student's performance and thus alleviated her anxiety. P17's discussion with colleagues about her student's difficulties in doing research helped her not only unburden herself but also obtain suggestions, which further led to situation modulation in the form of adjusting her supervision practice. On the other hand, the fact that the participants reached out for extrinsic emotion regulation underscores the emotion burdens of supervisors. Such tendency also highlights the need for all stakeholders to pay due attention to supervisors' psychological well-being along with doctoral students' emotions. As P13 rightly appealed: “Students can access in-house counseling service […] but we supervisors need it too.”

In addition, eight supervisors were found to suppress their anger and anxiety. Despite the prevalence of suppression found in Taxer and Gross (2018), this strategy was rarely documented in doctoral supervision research (except Halse, 2011). Our finding indicates that doctoral supervisors, at least in the disciplinary and institutional contexts of the current study, tended to suppress their emotions on spot, probably because some held the belief that displaying negative emotions would ruin supervisory relationship or shaken students' delicate confidence (P3 and P12). However, as habitual use of suppression may be maladaptive and even threatens health (Gross, 2015), the finding suggests that further investigation of supervisors' emotion regulation strategies and their short-term and long-term effects are needed.

## Limitation and Future Research Directions

The current study has a few limitations, such as small sample size, the exclusive focus on one discipline (i.e., computer science), single data source, and the absence of longitudinal data. Contextual limitations also exist, as the supervisors recruited were elite professors in Chinese higher education institutes. Moreover, some emotional experiences may be evanescent or occur at the sub-conscious level, so that they might not be readily verbalized.

However, the current findings can offer a few implications for future research. Greater research effort should be made to explore supervisors' vis-a-vis supervisees' emotional experiences to paint a more complete picture of the emotional dimension of doctoral supervision, as well as to enrich our knowledge about the factors mediating their emotional experiences. Given that supervisors utilized various emotion regulation strategies, it is worth investigating why and how they chose particular strategies on spot. Another issue to be examined is the effects of both positive and negative emotions and emotion regulation strategies on doctoral supervision, students' completion of degree, and even doctoral supervisors' own development. Finally, since the present study only looked into intrinsic emotion regulation of supervisors, investigations can be extended to understanding of how both parties regulate each other's emotions and the effectiveness of these endeavors.

## Recommendations for Doctoral Supervision Stakeholders

In terms of practical implications, the current inquiry stresses the need for stakeholders to pay close attention to supervisors' emotional experiences and psychological well-being, ideally in tandem with their students'. Institutions should provide supervisors with resources, such as in-house counseling service and workshops, to help them regulate their own emotions and improve their psychological well-being. Supervisors should carefully select students to minimize possible mismatch in working styles and beliefs; however, they should be aware that being selective in recruitment does not completely pre-empt all emotion-draining experiences. Rather, they should be aware of the existence of remarkable dissonance between their expectations and their students', and more importantly, to make rules and conventions explicit. In addition, supervisors themselves should develop their competence in emotion regulation in order to identify and regulate negative emotions at an early stage, so as not to let them aggregate and incur unfavorable consequences. Finally, supervisors can leverage on the power of community by communicating with colleagues and other trusted parties to enhance their emotional experiences.

## Conclusion

Based on individual interviews with 17 computer science professors, the qualitative study explored doctoral supervisors' emotions and their strategies to regulate those emotions. The data revealed their wide-ranging emotional experiences, with negative emotions being more frequent and diverse. The participants also employed various emotion regulation strategies, encompassing both antecedent-focused and response-focused approaches.

This study is one of the initial studies exploring doctoral supervisors' emotion and emotion regulation in their own right. The prevalence and diversity of supervisors' negative emotions, underscores the emotion-taxing challenges inherent to doctoral supervision (Aitchison et al., 2012), expands our knowledge about the antecedents of supervisors' negative emotions, and reveals the complex relationship between multiple emotions themselves. These insights collectively suggest that doctoral supervision as a cognitive as well as emotional endeavor. Another contribution of the study is uncovering various intrinsic emotion regulation strategies that supervisors utilize, especially the underreported strategies such as cognitive change and seeking extrinsic emotion regulation. Additionally, the findings can enrich the framework of emotion regulation strategies: Cognitive change can be applied to reappraising emotions themselves, in addition to reappraising antecedents of emotions; and that one can proactively seek extrinsic emotion regulation to improve one's own emotions.

## Data Availability Statement

The raw data supporting the conclusions of this article will be made available by the authors, without undue reservation.

## Ethics Statement

The studies involving human participants were reviewed and approved by Guangdong University of Foreign Studies. The patients/participants provided their written informed consent to participate in this study.

## Author Contributions

YH is mainly in charge of research methodology and drafting of the manuscript. YX is responsible for data collection and revision of the manuscript. All authors contributed to the article and approved the submitted version.

## Conflict of Interest

The authors declare that the research was conducted in the absence of any commercial or financial relationships that could be construed as a potential conflict of interest.

## Appendix

### Interview Guide

Thank you very much for making the time to participate in this study. We are going to have a 30-min interview, which aims to understand your emotional experience as a doctoral supervisor and your emotional regulation practice. There are no right or wrong answers to these questions. Please answer them based on your own experience.

- Please briefly introduce yourself.
- Please briefly describe your experience in supervising doctoral students.
- What emotions do you often experience as a doctoral supervisor?
- What do you think of your emotional experiences induced by doctoral supervision?
- Do you regulate your own emotions induced by doctoral supervision? If so, how? If not, why?
- What are the common issues and difficulties that you encounter in doctoral supervision?
- Please describe a moment/incident/event that strikes you in your doctoral supervision career. How did you feel then? Looking back at the moment/incident/event, how do you feel now?
- What advice would you give novice doctoral supervisors regarding the emotional dimension of doctoral supervision?
- Do you have further comments, reflections, and opinions about emotional experiences and emotion regulation of doctoral supervisors?
